# Trends in management and outcomes of COPD patients in primary care, 2000–2009: a retrospective cohort study

**DOI:** 10.1038/npjpcrm.2014.15

**Published:** 2014-07-03

**Authors:** Gareth D James, Gavin C Donaldson, Jadwiga A Wedzicha, Irwin Nazareth

**Affiliations:** 1 Department of Primary Care & Population Health, UCL Medical School, Royal Free Campus, Hampstead, London, UK; 2 Centre for Respiratory Medicine, University College London, Royal Free Campus, Hampstead, London, UK

## Abstract

**Background:**

Since the introduction of the Quality and Outcomes framework, there has been some evidence of improvement in the management of chronic obstructive pulmonary disease (COPD) patients in the United Kingdom through increasing rates of smoking cessation advice and immunisations against influenza. However, it is unknown whether disease-specific management criteria, disease outcomes and diagnosis have improved.

**Aims:**

To describe changes in the management and outcomes of patients with COPD in UK general practice between 2000 and 2009.

**Methods:**

The study was done on a retrospective cohort using data from The Health Improvement Network UK primary care database. We calculated age at diagnosis of COPD and death, total number of short-term oral corticosteroid courses and consultations, and proportion of patients with very severe COPD and on triple inhaled therapy for each year between 2000 and 2009.

**Results:**

We identified 92,576 patients with COPD. The mean age at COPD diagnosis decreased from 68.1 years in 2000 to 66.7 years in 2009. The mean age at death increased from 78.2 years in 2000 to 78.8 years in 2009. The number of prescribed courses of oral corticosteroids increased from 0.6 in 2000 to 0.8 in 2009. The number of consultations increased from 9.4 in 2004 to 11.3 in 2009. The risk of having very severe COPD decreased from 9.4% in 2004 to 6.8% in 2009. The likelihood of patients with very severe COPD receiving triple therapy increased from 25% in 2004 to 59% in 2009.

**Conclusions:**

The trends suggest that management and outcomes observed in patients with COPD may have improved since the year 2000.

## Introduction

Chronic obstructive pulmonary disease (COPD) has a substantial burden on individual patients and health services worldwide.^1^ Patients suffer breathlessness, exacerbations and reduced quality of life.^2^ In the UK, there are an estimated 900,000 people with COPD, and the disease costs the National Health Service (NHS) over £253 million each year.^3^

There have been a number of initiatives that should have led to continuous improvements in the spirometric assessment and management of patients with COPD in General Practice.^4^ Possibly, the most important was the Quality and Outcomes Framework in 2004, which is a voluntary incentive scheme for general practices in the UK that rewards patient care. Since its introduction, UK practices have continued to increase their achievement of Quality and Outcomes Framework indicators.^5^ Practices are paid if they demonstrate a register of patients with COPD and that these patients have spirometry performed and inhaler technique checked in the preceding 15 months and were given influenza immunisation. Other initiatives include publication of COPD management guidelines by the National Institute for Health and Clinical Excellence guidelines.^2^ Continuing professional development would help increase awareness of these initiatives. There is some evidence of improvement through increasing rates of smoking cessation advice^6^ and immunisations against influenza.^7^ However, it is not known whether disease-specific management criteria and disease outcomes of COPD patients have changed over time.

Early diagnosis of COPD was a specific aim of the National Institute for Health and Clinical Excellence guidelines. There has been some progress,^4^ but two-thirds of the UK population are unaware of the term COPD^8^ and thus may not seek help as they confuse the symptoms with age-related disability. Good management should involve regular patient–clinician consultations, prescription of inhaled therapy and encouragement to seek treatment for exacerbations when short courses of oral corticosteroids may be required.^2,9^ Triple inhaled therapy, the combination of a long-acting β_2_-agonist (LABA), a long-acting muscarinic antagonist (anticholinergic) bronchodilator (LAMA), and an inhaled corticosteroid (ICS) inhaler, has received much interest recently,^10,11^ but further evidence from randomised controlled trials is required to further quantify their efficacy and to determine which patient population they should be prescribed to. The National Institute for Health and Clinical Excellence guidelines recommend this therapy is provided to patients with persistent exacerbations or breathlessness despite treatment with a LAMA or LABA+ICS combination.

Using data from The Health Improvement Network (THIN) primary care database, we aimed to describe trends in the management and outcomes of patients with COPD in UK general practice since the year 2000. Management was assessed by age at diagnosis of COPD and death, and total number of consultations and short-term oral corticosteroid courses prescribed. We also examined trends in the proportion of patients with very severe COPD and on triple therapy, as these impose a large financial burden on health services and knowledge of any increase will be useful for heath-service planning.

## Materials and Methods

### Data source

In the UK, general practitioners (GPs) record medical diagnosis and symptoms, health indicators such as smoking status and prescribed medicines during consultations with patients. In all, 98% of the population is registered with a GP^12^ and COPD is primarily managed in general practice. The THIN primary care database includes anonymised medical and prescribing records from 6% of the UK population (details concerning the number of practices and active patients can be found at http://csdmruk.cegedim.com/our-data/statistics.shtml). The database is broadly representative of UK general practice in terms of gender, age and smoking status,^13,14^ and it has been validated for pharmacoepidemiological research.^15^ Recorded diagnoses and symptoms are classified using Read codes.^16^ Prescription data are comparable with national statistics,^17^ and over 98% of prescriptions are redeemed.^18^ The database includes quintiles of the Townsend score,^19^ a composite measure of social deprivation (occupation, unemployment, car ownership, overcrowding) based on data from the 2001 Census and mapped to a patient’s postal code. The fifth quintile is most deprived and the first quintile is least deprived. Accurate data recording in THIN is ensured by only including practices newly involved in THIN when they meet acceptable recording standards and practice standardised mortality rates match externally collected data from the National death registration statistics.^20^ The prevalence, demographics, smoking habits and mortality of patients with COPD in the database match national data.^4^

### Study population

We included all patients who were aged between 35 and 89 years registered in THIN between 2000 and 2009. Patients entered the study on the latest date of either their 35th birthday or 1 January 2000, when they registered with the practice or the practice met the quality standard for data recording. Data were collected until the earliest date of their 90th birthday, death, transfer to another practice or 31 December 2009.

Our sample of COPD patients included all eligible patients with a diagnosis of COPD based on the Quality and Outcomes Framework Read codes for COPD. These codes have been shown to be helpful in selecting a subset of patients whose prevalence, geographical distribution, age and gender distribution match other national COPD surveys^4^ and have been used in other COPD studies.^21,22^ Patients were considered to have the disease from the date of diagnosis—although the patients may have had the disease for some time before.

We extracted information on the latest recorded smoking status (active/non/ex-smoker), height and quintiles of Townsend deprivation scores for all patients. We also extracted the forced expiratory volume in 1 s (FEV_1_) and forced vital capacity values (FVC) before 2004 and annual measurements between 2004 and 2009. When information on FEV_1_ was unavailable, it was carried forward from previous years. FEV_1_ and FVC measurements outside a 0.3–7.0 litre range were considered invalid. All oral corticosteroid prescriptions of ⩽14 days and prescriptions of a LAMA (tiotropium), LABA (salmeterol or formoterol), ICS (fluticasone or budesonide), LABA+ICS combination (salmeterol + fluticasone or formoterol + budesonide) and medical diagnosis records were extracted for all patients with COPD from their date of diagnosis. Oral corticosteroids were used as a proxy for exacerbations. Triple therapy was defined as co-prescriptions of a LAMA (tiotropium) and an ICS/LABA combination inhaler (either salmeterol + fluticasone (Seretide), or formoterol + budesonide (Symbicort)). Prescriptions of these compounds with three separate inhalers were also included in case the combination was recorded separately. A third combination therapy, Fostair, had not received a UK license by 2009. Tiotropium was not available until 2002 so annual estimates were only calculated from 2004 onwards. We are aware that there are other ICSs, but we did not examine these as they are not part of the LABA + ICS combination inhalers that constitute triple therapy.

The criteria devised by Global Initiative for Chronic Obstructive Lung Disease were used to classify the severity of disease.^9^ Predicted FEV_1_ was estimated using age, sex and height, using the method in Quanjer *et al.* ^23^ Severity was classified as follows: ‘mild’ if FEV_1_⩾80% predicted, ‘moderate’ if FEV_1_ ⩾50% and <80% predicted, ‘severe’ if FEV_1_ ⩾30% and <50% predicted, and ‘very severe’ if FEV_1_ <30% predicted.

### Management and outcome indices

We considered six indices of management and outcomes: age at diagnosis and death, total number of short-term oral corticosteroid courses and consultations, the proportion of patients with very severe COPD and the proportion of patients with very severe COPD on triple therapy. The indices were calculated for patients with COPD in each calendar year.

The age of death was calculated for all patients and all COPD patients who had died at the age of ⩾65 years, as below this age deaths are unlikely to be tobacco related. The age of patients with COPD in each calendar year was also calculated.

Spirometry was not well recorded before 2004, and thus the severity grade by the Global Initiative for Chronic Obstructive Lung Disease classification was only calculated from 2004 onwards. The proportion of patients in each category was then calculated for each calendar year. A patient consultation was identified as a single day with one or more medical diagnosis codes for COPD recorded by the patient’s GP.

### Statistical analysis

The characteristics of patients with COPD and the whole population were tabulated. The annual prevalence of COPD was calculated by dividing the total follow-up time contributed by the COPD population by the sum of the follow-up time of the whole population within a given year. Annual incidence rates of COPD were calculated by dividing the total number of newly diagnosed COPD patients by the sum of the whole population follow-up time within a given year. Follow-up time (patient-years) was preferred to number of patients as it takes account of the amount of time each patient is alive or registered with their practice. The mean and corresponding 95% confidence intervals (CIs) were calculated for each quality of care index in 2000, 2004 and 2009.

We assessed changes over time in the management and outcome indices. Mean age at diagnosis and death between 2000 and 2009 were assessed using linear regression. Poisson generalised estimating equations were constructed with unstructured covariance to estimate the annual rate of change in the number of oral corticosteroids and consultations per patient-year between 2000 and 2009 and the probability of a patient with very severe COPD receiving triple therapy in each calendar year between 2004 and 2009. The generalised estimating equation method was chosen to account for repeated measurements within patients. A logistic model was constructed to estimate the probability of having very severe COPD in 2004 and 2009 relative to any other severity classification. All models were constructed without adjustment, and once again with adjustment for age, sex and Townsend quintile.

All statistical analyses were conducted using Stata version 12.0 (Stata, College Station, TX, USA).

### Ethics

The THIN scheme for obtaining and providing anonymous patient data to researchers was approved by the National Health Service South-East Multicenter Research Ethics Committee (MREC) in 2002.

## Results

### Patient characteristics

**Figure 1:**
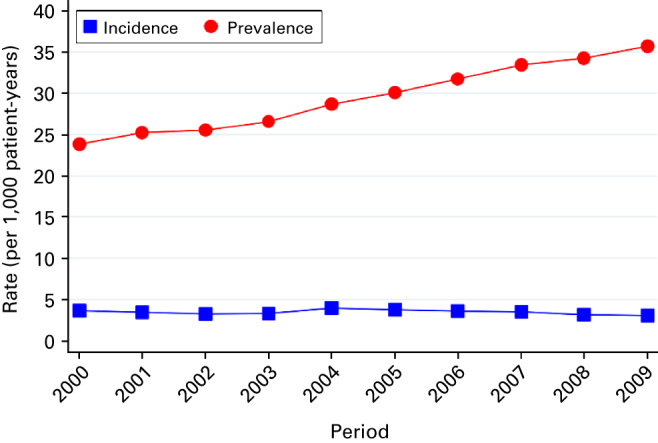
Prevalence and incidence rates of chronic obstructive pulmonary disease between 2000 and 2009.

In total, there were 2,839,694 patients in 419 general practices who were aged between 35 and 89 years between 2000 and 2009; 92,576 (3%) had a clinical record of COPD. The average follow-up time from COPD diagnosis was 3 years and 10 months. The mean age of prevalent COPD patients was 71 years, and 54% of patients were male. In comparison with the general population, patients with COPD were older and from more socially deprived areas (Table 1). The recorded prevalence of COPD increased from 24 individuals per 1,000 patient-years in 2000 to 36 in 2009, equivalent to a 50% increase over the 10-year period (Figure 1). However, the diagnosis of new COPD cases remained fairly constant at 3.5 individuals per 1,000 patient-years.

For the COPD population, data were 100% complete for age and sex, 95% for deprivation data as measured by Townsend quintiles, 91% for height and smoking status, 72% for FEV_1_ values and 52% for FVC values. Annual FEV_1_ recording increased each year, from 47% of patients in 2004 to 81% in 2009.

### Summary measures for the management and outcome indices

**Figure 2:**
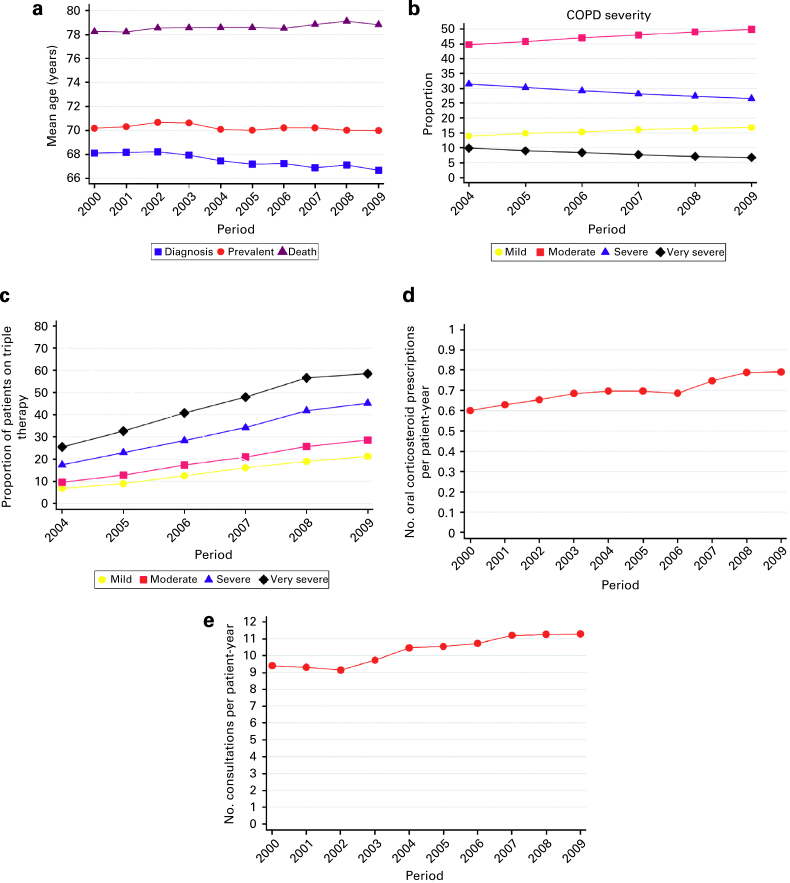
Management and outcome indices in chronic obstructive pulmonary disease (COPD): 2000–2009. (**a**) Mean age of prevalent COPD cases, at COPD diagnosis and at death. (**b**) Proportion of patients in each COPD severity category. (**c**) Proportion of COPD patients who received triple therapy. (**d**) Number of oral corticosteroid prescriptions per COPD patient-year. (**e**) Number of COPD consultations per patient-year.

The mean age at which COPD was diagnosed decreased from 68.1 years (95% CI, 67.8, 68.4) in 2000 to 66.7 years in 2009 (66.4, 67.0; Table 2 and Figure 2a). The mean age at death of COPD patients increased from 78.2 years (77.9, 78.6) in 2000 to 78.8 years (78.6, 79.1) in 2009. Similarly, the mean age at death of all patients increased from 79.5 years (79.3, 79.6) in 2000 to 80 years (79.9, 80.1) in 2009. Consequently, the mean age of prevalent COPD patients remained fairly constant over the period, varying between 70 and 70.7 years.

In 2004, 10% of patients had ‘very severe’ COPD, as defined by the Global Initiative for Chronic Obstructive Lung Disease spirometric criteria,^9^ and this decreased to 7% in 2009 (Table 2 and Figure 2b). The use of triple inhaled therapy between 2004 and 2009 increased from 7 to 21% in mild COPD, from 10 to 29% in moderate COPD, from 17 to 45% in severe COPD and from 25 to 59% in very severe COPD (Table 2, Figure 2c).

In total, 462,301 courses of oral corticosteroids were prescribed to patients with COPD between 2000 and 2009. The mean number of oral corticosteroid prescriptions per patient-year increased from 0.6 (95% CI, 0.6, 0.6) in 2000 to 0.8 (0.8, 0.8) in 2009 (Table 2 and Figure 2d).

The COPD patients attended 3,688,949 consultations between 2000 and 2009. The mean number of consultations per patient-year increased from 9.4 (9.4, 9.4) in 2000 to 11.3 (11.3, 11.3) in 2009 (Table 2 and Figure 2e).

### Hypothesis testing for the management and outcome indices

There was little difference in the regression coefficients for annual change between the unadjusted models and the models adjusted for age, sex and Townsend quintile (Tables 3a and b). Between 2000 and 2009, each additional year was associated with a 0.18-year decrease (95% CI, 0.15, 0.21) in the mean age at diagnosis, a 0.07-year increase (0.04, 0.10) in the mean age at death, a 4.9% (4.7%, 5.1%) increase in the number of prescribed oral corticosteroids and a 2.8% (2.8%, 2.9%) increase in the number of consultations. Patients had 0.67 (0.64, 0.72) times the risk of having very severe COPD in 2009 compared with 2004. Between 2004 and 2009, the likelihood of very severe COPD patients receiving triple therapy increased by 15% (14%, 17%) each year.

## Discussion

### Main findings

This study provides evidence that the management and outcomes of patients with COPD in UK primary care has changed between 2000 and 2009. GPs are identifying COPD earlier, and patients with the disease are being monitored more regularly and treated more in accordance with the recent National Institute for Health and Clinical Excellence guidelines. These improvements may have contributed to the improved longevity and reduced severity of disease in the COPD patients we observed.

### Interpretation of findings in relation to previously published work

The estimate of COPD prevalence obtained from this study is considerably lower than that obtained by conducting spirometry on the general population, indicating that COPD may be underdiagnosed in primary care. This study and others^24–27^ have found that the prevalence of COPD is increasing, which may indicate that small improvements in diagnosis are being made. Our sample does not represent the prevalence of COPD in the community as defined by FEV_1_ values, as many people may have the disease on this basis but are not diagnosed because their respiratory symptoms are mild. Notably, in terms of symptomatic COPD, Shahab *et al.* ^24^ found 2.7% of the general population presented with COPD in 2004, which is similar to our prevalence estimate. Smith *et al.* ^4^ also reported that the prevalence of COPD in UK general practice was considerably lower than that obtained using spirometry and that the prevalence increased between 2003 and 2005. The increase in prevalence of COPD and the total number of consultations made by these patients corresponds to COPD having an increasing burden on general practice.

COPD patients have benefited from small improvements in longevity, similar to that seen in the general population.^28^ It is likely that this is due to better public health advice and improvements in health care that are as good as those seen in the other major causes of death, and that most COPD patients die from co-morbidities. Earlier diagnosis of COPD is an important finding, as early intervention may control symptoms, improve the quality of life and reduce the impact of exacerbations.^8^ Increased awareness of COPD in recent years has been noted in other studies^4^ and may partly explain the earlier identification of COPD.

The proportion of patients with severe and very severe COPD in the primary care population has decreased over time. The carrying forward of FEV_1_ records from previous years does not explain this decrease, as the same analysis using the raw data indicates an additional 1% fall in the proportion of patients with severe and very severe COPD. The decreasing severity could be due to an expansion in the COPD population of mild and moderate disease through earlier detection and diagnosis, or improved therapy preventing disease progression. We observed a marked increase in the use of triple therapy. As expected, patients with more severe COPD have experienced a larger increase in the use of triple therapy in accordance with current guidelines.^9^ We also found that up to a third of patients with mild or moderate COPD severity were on triple therapy, which is outside current recommendations unless the patients experience frequent exacerbations. The ECLIPSE observational study has reported that 22% of patients worldwide with moderate COPD severity would be categorised as a frequent exacerbator, as they suffer two or more exacerbations per year.^29^ Although we cannot be certain from our data, it is possible that the patients we observed with mild or moderate COPD severity who were prescribed triple inhaled therapy are perhaps being treated as per the current guidelines.

Given the expansion in the proportion of patients with mild and moderate COPD, it is unlikely that the mean number of exacerbations per patient has increased over time.^30^ The rise in the number of oral corticosteroids per year could be explained by patients being encouraged to seek GP help when they have an exacerbation or the greater likelihood of GPs prescribing oral corticosteroids for exacerbations.

### Strengths and limitations of this study

This study has many strengths. The data were large (THIN comprises 6% of the UK population), longitudinal and generalisable to the UK general practice population and contained information on patient characteristics, diagnosis and prescriptions, which allowed us to assess many measures of quality of care over time. In addition, we used validated codes for identifying COPD patients. The study, however, was limited by the lack of information on hospitalisations and exacerbations, which would have provided a more comprehensive picture of changes in patient management. In addition, it may be advantageous to have investigated triple therapy involving ICS agents other than fluticasone or budesonide, but this would have been considerably more complicated. We also did not assess whether triple therapy was appropriately prescribed or not. A further limitation is the use of oral corticosteroid prescriptions as a proxy for exacerbations, as a large proportion of exacerbations may be treated with antibiotics alone. However, we considered oral corticosteroids to provide greater sensitivity, as antibiotics can be prescribed for many reasons other than a pulmonary infection. It is also possible that changes in the management of co-morbid conditions may have influenced the results, for example, improvements in the care of cardiovascular disease may have improved the longevity of COPD patients, whereas the increased incidence of diabetes in the UK population may have had the reverse effect.

There will be a number of reasons for the differences in the management of COPD patients in UK general practice observed in this study and other studies,^5–7^ including the Quality and Outcomes Framework, clearer management guidelines, professional development and recognition of the need to screen patients for this disease. Their implementation and effect would overlap, and therefore it was not possible to ascribe improvements to any single initiative. It is not yet known whether setting clearer guidelines for appropriate management will improve outcomes such as mortality.

### Implications for future research, policy and practice

Increasing awareness of COPD and management initiatives have changed the outcomes in COPD. Current and any new initiatives should continue to be supported.

## Conclusion

Longitudinal trends since the year 2000 suggest that patients with COPD are being diagnosed, assessed and treated more. This might partly explain why COPD patients appear to be living longer and have reduced severity of disease.

## Figures and Tables

**Table 1:** Characteristics of patients with COPD and the whole population

*Variable*	*Category*	*All*	*COPD*
Total patient no.	—	2,839,694	92,576
Age^a^ (mean, years)	—	54.8	70.5
Age^a^ (%)	35–39	21.4	0.6
	40–49	24.2	3.8
	50–59	19.8	12.9
	60–69	15.0	27.3
	70–79	11.5	33.8
	80–89	8.2	21.6
Gender (%)	Male	49.1	53.8
	Female	50.9	46.2
Smoking (%)	Non	53.2	14.4
	Active	21.2	32.6
	Ex	25.7	53.0
Townsend quintile (%)	1 (least deprived)	27.1	16.5
	2	22.8	18.1
	3	20.8	21.3
	4	17.6	23.8
	5 (most deprived)	11.8	20.3
FEV_1_ (mean, l)	—	—	1.4
FVC (mean, l)	—	—	2.4

Abbreviations: COPD, chronic obstructive pulmonary disease; FEV_1_, forced expiratory volume in 1 s; FVC, forced vital capacity.

a Age was determined at the mid-point of each patient’s observation period.

**Table 2:** Management and outcome indices in 2000, 2004 and 2009 for COPD and all patients

*Outcome*	*2000*		*2004*		*2009*	
*COPD patients*	N	*Mean (95% CI)*	N	*Mean (95% CI)*	N	*Mean (95% CI)*
Age at diagnosis (years)	5,993	68.1 (67.8, 68.4)	7,834	67.4 (67.2, 67.7)	5,774	66.7 (66.5, 66.7)
Age at death (years)	1,195	78.2 (77.9, 78.6)	2,178	78.6 (78.3, 78.8)	2,582	78.8 (78.6, 79.1)
						
	N	*% (95% CI)*	N	*% (95% CI)*	N	*% (95% CI)*
Very severe COPD (%)	—	—	19,034	9.9 (9.5, 10.3)	45,654	6.8 (6.6, 7.0)
*Triple therapy (%)*						
Mild COPD	—	—	2,650	6.7 (5.8, 7.7)	7,668	21.1 (20.2, 22.1)
Moderate COPD	—	—	8,515	9.5 (8.9, 10.1)	22,747	28.6 (28.0, 29.1)
Severe COPD	—	—	5,985	17.4 (16.4, 18.4)	12,137	45.2 (44.3, 46.1)
Very severe COPD	—	—	1,884	25.4 (23.5, 27.5)	3,094	58.6 (56.8, 60.3)
						
	N	*Per patient (95% CI)*	N	*Per patient (95% CI)*	N	*Per patient (95% CI)*
Oral corticosteroid prescriptions	16,851	0.6 (0.6, 0.6)	33,924	0.7 (0.7, 0.7)	49,203	0.8 (0.8, 0.8)
Consultations	16,851	9.4 (9.4, 9. 4)	33,924	10.5 (10.4, 10.5)	49,203	11.3 (11.3, 11.3)
						
*All patients*	N	*Mean (95% CI)*	N	*Mean (95% CI)*	N	*Mean (95% CI)*
Age at death (years)	10,569	79.5 (79.3, 79.6)	15,449	79.53 (79.4, 79.6)	16,179	80.0 (79.9, 80.1)

Abbreviations: CI, confidence interval; COPD, chronic obstructive pulmonary disease.

**Table 3:** Management and outcome indices hypothesis testing: (a) all patients with COPD; (b) all patients with very severe COPD

*Index*	*Adjustment*	*Period*		*Period, age, sex, townsend*	
		*Coef (95% CI)*	P*-value*	*Coef (95% CI)*	P*-value*
*(a)*					
Mean age at diagnosis: linear, *n*=65,900	Annual change	−0.18 (−0.21, −0.15)	<0.01	−0.19 (−0.22, −0.16)	<0.01
	Sex	—	—	0.21 (0.04, 0.38)	0.02
	Townsend 1	—	—	1.00	
	Townsend 2	—	—	−0.49 (−0.78, −0.19)	
	Townsend 3	—	—	−1.64 (−1.92, −1.35)	
	Townsend 4	—	—	−2.23 (−2.50, −1.95)	
	Townsend 5	—	—	−3.74 (−4.03, −3.45)	
	Townsend missing	—	—	−1.80 (−2.23, −1.37)	<0.01
					
Mean age at death: linear, *n*=20,932	Annual change	0.08 (0.04, 0.11)	<0.01	0.07 (0.04, 0.1)	<0.01
	Sex	—	—	0.56 (0.39, 0.73)	<0.01
	Townsend 1 (Ref)	—	—	1.00	
	Townsend 2	—	—	−0.19 (−0.48, 0.11)	
	Townsend 3	—	—	−0.48 (−0.77, −0.19)	
	Townsend 4	—	—	−1.03 (−1.3, −0.75)	
	Townsend 5	—	—	−1.5 (−1.79, −1.21)	
	Townsend missing	—	—	0.22 (−0.22, 0.67)	<0.01
					
Proportion of patients with very severe COPD: odds ratio, *n*=52,311	Annual change	0.66 (0.62, 0.70)	<0.01	0.67 (0.64, 0.72)	<0.01
	Age	—	—	0.99 (0.99, 1.00)	<0.01
	Sex	—	—	0.52 (0.48, 0.55)	<0.01
	Townsend 1 (Ref)	—	—	1.00	
	Townsend 2	—	—	0.97 (0.88, 1.08)	
	Townsend 3	—	—	1.01 (0.91, 1.11)	
	Townsend 4	—	—	1.09 (0.99, 1.20)	
	Townsend 5	—	—	1.10 (1.00, 1.22)	
	Townsend missing	—	—	0.99 (0.85, 1.17)	0.03
					
No. of oral corticosteroids: incident rate ratio, *n*=92,576	Annual change	1.05 (1.05, 1.05)	<0.01	1.05 (1.05, 1.05)^a^	<0.01
	Age	—	—	1.01 (1.01, 1.01)	<0.01
	Sex	—	—	1.21 (1.20, 1.23)	<0.01
	Townsend 1 (Ref)	—	—	1.00	
	Townsend 2	—	—	1.03 (1.01, 1.06)	
	Townsend 3	—	—	1.07 (1.05, 1.10)	
	Townsend 4	—	—	1.12 (1.10, 1.15)	
	Townsend 5	—	—	1.20 (1.18, 1.23)	
	Townsend missing	—	—	1.08 (1.05, 1.12)	<0.01
					
No. of consultations: incident rate ratio, *n*=92,576	Annual change	1.03 (1.03, 1.03)	<0.01	1.03 (1.03, 1.03)^b^	<0.01
	Age	—	—	1.01 (1.01, 1.01)	<0.01
	Sex	—	—	1.08 (1.08, 1.08)	<0.01
	Townsend 1 (Ref)	—	—	1.00	
	Townsend 2	—	—	0.99 (0.98, 1.00)	
	Townsend 3	—	—	1.03 (1.03, 1.04)	
	Townsend 4	—	—	1.01 (1.01, 1.02)	
	Townsend 5	—	—	1.01 (1.00, 1.01)	
	Townsend missing	—	—	1.09 (1.08, 1.10)	<0.01

*(b)*					
Proportion of patients receiving triple therapy: incident rate ratio, *n*=6,456	Annual change	1.15 (1.14, 1.17)	<0.01	1.16 (1.14, 1.18)	0.00
	Age	—	—	0.99 (0.99, 0.99)	0.00
	Sex	—	—	0.94 (0.88, 1.01)	0.09
	Townsend 1 (Ref)	—	—	1.04 (0.92, 1.16)	
	Townsend 2	—	—	1.06 (0.95, 1.19)	
	Townsend 3	—	—	1.07 (0.96, 1.19)	
	Townsend 4	—	—	1.03 (0.92, 1.15)	
	Townsend 5	—	—	0.98 (0.82, 1.17)	
	Townsend missing	—	—	1.16 (1.14, 1.18)	0.77

Abbreviations: CI, confidence interval; Coef, coefficient; COPD, chronic obstructive pulmonary disease.

a 1.049 (1.047, 1.051) to 3 dp.

b 1.028 (1.028, 1.029) to 3 dp.

## Notes

The authors declare no conflict of interest. This study was designed and analysed independent of any input from the National Institute of Health Research, who were the funders. No additional data were available. All authors have completed the Unified Competing Interest form and declare no support from any organisation for the submitted work; no financial relationships with any organisations that might have an interest in the submitted work in the previous 3 years; and no other relationships or activities that could appear to have influenced the submitted work. The views expressed are those of the author(s) and not necessarily those of the NHS, the NIHR or the Department of Health.

## References

[bib1] Rabe KF Wedzicha JA Controversies in treatment of chronic obstructive pulmonary disease Lancet 2011 378 1038 1047 21907867 10.1016/S0140-6736(11)61295-6

[bib2] National Clinical Guideline Centre Chronic Obstructive Pulmonary Disease: Management of Chronic Obstructive Pulmonary Disease in Adults in Primary and Secondary Care National Clinical Guideline Centre: London, UK 2010 . Available from http://guidance.nice.org.uk/CG101/Guidance/pdf/English .

[bib3] British Thoracic Society , 2nd edn, 2006. Available from http://www.brit-thoracic.org.uk/Portals/0/Library/BTS%20Publications/burdeon_of_lung_disease2007.pdf .

[bib4] Smith CJ Gribbin J Challen KB Hubbard RB The impact of the 2004 NICE guideline and 2003 General Medical Services contract on COPD in primary care in the UK QJM 2008 101 145 153 18180254 10.1093/qjmed/hcm155

[bib5] Doran T Fullwood C Kontopantelis E Reeves D Effect of financial incentives on inequalities in the delivery of primary clinical care in England: analysis of clinical activity indicators for the quality and outcomes framework Lancet 2008 372 728 736 18701159 10.1016/S0140-6736(08)61123-X

[bib6] Taggar JS Coleman T Lewis S Szatkowski L The impact of the Quality and Outcomes Framework (QOF) on the recording of smoking targets in primary care medical records: cross-sectional analyses from The Health Improvement Network (THIN) database BMC Public Health 2012 12 329 22559290 10.1186/1471-2458-12-329 PMC4104830

[bib7] Kontopantelis E Doran T Gravelle H Goudie R Siciliani L Sutton M Family doctor responses to changes in incentives for influenza immunization under the U.K. Quality and Outcomes Framework pay-for-performance scheme Health Serv Res 2012 47 (Pt 1) 1117 1136 22171997 10.1111/j.1475-6773.2011.01362.x PMC3423175

[bib8] Britton M The burden of COPD in the U.K.: results from the confronting COPD survey Respir Med 2003 97 (Suppl C) S71 S79 12647945 10.1016/s0954-6111(03)80027-6

[bib9] Global Initiative for Chronic Obstructive Lung Disease

[bib10] Aaron S Vandemheen K Fergusson D Maltais F Bourbeau J Goldstein R Tiotropium in combination with placebo, salmeterol, or fluticasone–salmeterol for treatment of chronic obstructive pulmonary disease Ann Intern Med 2007 146 545 555 17310045 10.7326/0003-4819-146-8-200704170-00152

[bib11] Welte T Miravitlles M Hernandez P Eriksson G Peterson S Polanowski T Efficacy and tolerability of budesonide/formoterol added to tiotropium in patients with chronic obstructive pulmonary disease Am J Resp Crit Care Med 2009 180 741 750 19644045 10.1164/rccm.200904-0492OC

[bib12] Lis Y Mann RD The VAMP Research multi-purpose database in the U.K. J Clin Epidemiol 1995 48 431 443 7897464 10.1016/0895-4356(94)00137-f

[bib13] Blak BT Thompson M Dattani H Bourke A Generalisability of The Health Improvement Network (THIN) database: demographics, chronic disease prevalence and mortality rates Inform Prim Care 2011 19 251 255 22828580 10.14236/jhi.v19i4.820

[bib14] Szatkowski L Lewis S McNeill A Huang Y Coleman T Can data from primary care medical records be used to monitor national smoking prevalence? J Epidemiol Community Health 2012 66 791 795 21571750 10.1136/jech.2010.120154

[bib15] Lewis JD Schinnar R Bilker WB Wang X Strom BL Validation studies of the health improvement network (THIN) database for pharmacoepidemiology research Pharmacoepidemiol Drug Saf 2007 16 393 401 17066486 10.1002/pds.1335

[bib16] Chisholm J The Read clinical classification BMJ 1990 300 1092 2344534 10.1136/bmj.300.6732.1092 PMC1662793

[bib17] Bourke A Dattani H Robinson M Feasibility study and methodology to create a quality-evaluated database of primary care data Inform Prim Care 2004 12 171 177 15606990 10.14236/jhi.v12i3.124

[bib18] The NHS Information Centre Prescribing and Primary Care Services 2011 . The Health and Social Care Information Centre. http://www.hscic.gov.uk/catalogue/PUB01500/pres-comp-rev-prop-pres-disp-rep.pdf .

[bib19] Townsend P Phillimore P Beattie A Inequalities in Health in the Northern Region Northern Regional Health Authority and University of Bristol: Newcastle upon Tyne 1986

[bib20] Maguire A Blak BT Thompson M The importance of defining periods of complete mortality reporting for research using automated data from primary care Pharmacoepidemiol Drug Saf 2009 18 76 83 19065600 10.1002/pds.1688

[bib21] Donaldson GC Hurst JR Smith CJ Hubbard RB Wedzicha JA Increased risk of myocardial infarction and stroke following exacerbation of COPD Chest 2010 137 1091 1097 20022970 10.1378/chest.09-2029

[bib22] James GD Petersen I Nazareth I Wedzicha JA Donaldson GC Use of long-term antibiotic treatment in COPD patients in the UK: a retrospective cohort study Prim Care Respir J 2013 22 271 277 23839240 10.4104/pcrj.2013.00061 PMC6442816

[bib23] Quanjer PH Tammeling GJ Cotes JE Pedersen OF Peslin R Yernault JC Lung volumes and forced ventilatory flows. Report working party standardization of Lung Function Tests, European Community for Steel and Coal. Official Statement of the European Respiratory Society Eur Respir J Suppl 1993 16 5 40 8499054

[bib24] Shahab L Jarvis MJ Britton J West R Prevalence, diagnosis and relation to tobacco dependence of chronic obstructive pulmonary disease in a nationally representative population sample Thorax 2006 61 1043 1047 17040932 10.1136/thx.2006.064410 PMC2117062

[bib25] Dickinson JA MM Searle M Ratcliffe G Screening older patients for obstructive airways disease in a semi-rural practice Thorax 1999 54 501 505 10335003 10.1136/thx.54.6.501 PMC1745503

[bib26] Mannino DM Buist AS Global burden of COPD: risk factors, prevalence, and future trends Lancet 2007 370 765 773 17765526 10.1016/S0140-6736(07)61380-4

[bib27] Buist AS McBurnie MA Vollmer WM Gillespie S Burney P Mannino DM International variation in the prevalence of COPD (the BOLD Study): a population-based prevalence study Lancet 2007 370 741 750 17765523 10.1016/S0140-6736(07)61377-4

[bib28] Office for National Statistics Life expectancy at birth and at age 65 for health areas in the United Kingdom, 2003–05 to 2007–09 2011 . http://www.ons.gov.uk/ons/rel/subnational-health4/life-expectancy-at-birth-and-at-age-65-for-health-areas-in-the-united-kingdom/2003-05-to-2007-09/index.html .

[bib29] Hurst JR Vestbo J Anzueto A Locantore N Mullerova H Tal-Singer R Susceptibility to exacerbation in chronic obstructive pulmonary disease N Engl J Med 2010 363 1128 1138 20843247 10.1056/NEJMoa0909883

[bib30] Donaldson GC Mullerova H Locantore N Hurst JR Calverley PM Vestbo J Factors associated with change in exacerbation frequency in COPD Respir Res 2013 14 79 23899210 10.1186/1465-9921-14-79 PMC3733814

